# A Tactile Sensor Network System Using a Multiple Sensor Platform with a Dedicated CMOS-LSI for Robot Applications †

**DOI:** 10.3390/s17091974

**Published:** 2017-08-28

**Authors:** Chenzhong Shao, Shuji Tanaka, Takahiro Nakayama, Yoshiyuki Hata, Travis Bartley, Yutaka Nonomura, Masanori Muroyama

**Affiliations:** 1 Department of Robotics, Tohoku University, Miyagi 980-8579, Japan; tanaka@mems.mech.tohoku.ac.jp; 2 Microsystem Integration Center, Tohoku University, Miyagi 980-8579, Japan; tbartley@uci.edu (T.B.); muroyama@mems.mech.tohoku.ac.jp (M.M.); 3 Partner Robot Div., Toyota Motor Corporation, Toyota, Aichi 470-0309, Japan; takahiro_nakayama_aa@mail.toyota.co.jp; 4 System & Electronics Engineering Dept. III, Toyota Central R&D Labs., Inc., Nagakute, Aichi 480-1192, Japan; yhata@mosk.tytlabs.co.jp; 5 Department of Mechatronics Engineering, Meijo University, Nagoya, Aichi 468-8502, Japan; nonomura@meijo-u.ac.jp

**Keywords:** next generation robot, sensor platform LSI, multi-kind sensing, tactile sensor network system, multi-point bus connection, single-ended communication

## Abstract

Robot tactile sensation can enhance human–robot communication in terms of safety, reliability and accuracy. The final goal of our project is to widely cover a robot body with a large number of tactile sensors, which has significant advantages such as accurate object recognition, high sensitivity and high redundancy. In this study, we developed a multi-sensor system with dedicated Complementary Metal-Oxide-Semiconductor (CMOS) Large-Scale Integration (LSI) circuit chips (referred to as “sensor platform LSI”) as a framework of a serial bus-based tactile sensor network system. The sensor platform LSI supports three types of sensors: an on-chip temperature sensor, off-chip capacitive and resistive tactile sensors, and communicates with a relay node via a bus line. The multi-sensor system was first constructed on a printed circuit board to evaluate basic functions of the sensor platform LSI, such as capacitance-to-digital and resistance-to-digital conversion. Then, two kinds of external sensors, nine sensors in total, were connected to two sensor platform LSIs, and temperature, capacitive and resistive sensing data were acquired simultaneously. Moreover, we fabricated flexible printed circuit cables to demonstrate the multi-sensor system with 15 sensor platform LSIs operating simultaneously, which showed a more realistic implementation in robots. In conclusion, the multi-sensor system with up to 15 sensor platform LSIs on a bus line supporting temperature, capacitive and resistive sensing was successfully demonstrated.

## 1. Introduction

Robots with tactile sense can recognize contacts with objects including touches from humans. Such robots also can distinguish materials with some criteria such as hardness, temperature and texture. Therefore, the tactile sense gives robots a better understanding of the surroundings, prevents robots from causing harm to humans, improves robots’ self-protection ability, and enriches human–robot communication.

To implement large-scale tactile sensors for covering robot bodies, one solution is to use a tactile sensor array with a scanning readout circuitry. To date, some scanning-readout type sensor arrays, which can include many sensing elements (capacitors or resistors), were reported for robot use, but the covering area is limited to several centimeters square [1,2,3,4,5]. With an increase in the number of sensing elements and the covering area, the number and length of wires increase. The interference effects along the long signal propagation pathway [6], the crosstalk from element circuits [7,8] and the long scanning time make this solution difficult to scale efficiently for large-scale artificial skins [9].

On the other hand, a serial bus-based tactile sensor network system has advantages in reducing the amount of wiring [10,11], the interference effects and the crosstalk because of the digitization of the sensor output on the site of sensation. However, the long sensor data conversion time of the analog-to-digital converter chip used by the conventional serial bus-based tactile sensor network system (8.73 ms and 1.6 ms for [10] and [11], respectively) limits the system performance [12].

**Figure 1:**
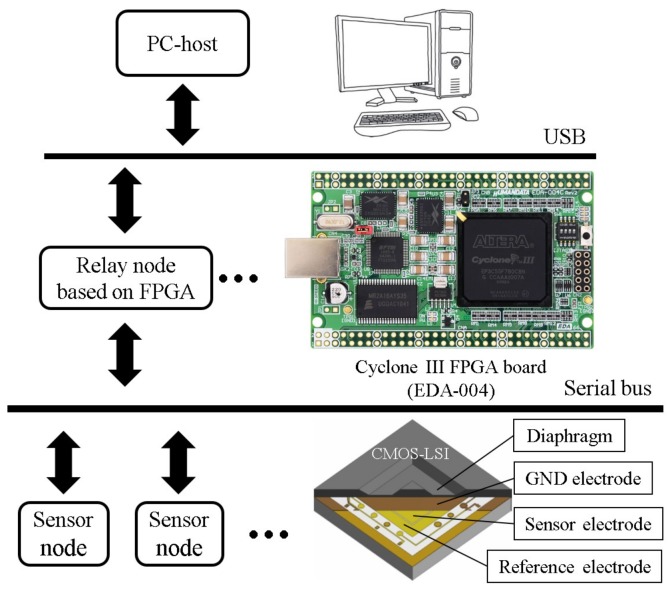
Serial bus-based tactile sensor network system composed of integrated capacitive type tactile sensors [14,15].

As shown in Figure 1, in our previous research [13], we have proposed a tactile sensor network system, including three network layers: sensor node, relay node and host layers. By using the integration and packaging technologies of a Micro-Electro-Mechanical-Systems (MEMS) capacitive sensor and a Complementary Metal-Oxide-Semiconductor (CMOS) Large-Scale Integration (LSI) circuit chip, we fabricated the sensor node, which has already been completed and evaluated at device and system levels [14,15]. Since the MEMS sensor is locally connected to a sensor readout circuit, this sensor system has high sensitivity, high noise tolerance and high speed response. For the capacitive sensor readout circuit, a capacitance-to-frequency converter and a frequency counter are embedded in the CMOS-LSI of the sensor node. Therefore, the capacitance information can be encoded into data packets as digital count values. The Field-Programmable Gate Array (FPGA) based relay node receives data packets from sensor nodes through a shared serial bus. After decoding, the relay node transmits these data packets to the PC-based host for analyzing through a Universal Serial Bus (USB) communication.

In the previous system, only capacitive tactile sensors were used. Since we used MEMS-CMOS integration and packaging technologies to fabricate the sensor node, the relationship between the external force and its decoded output becomes linear [16]. This linear relationship qualifies our sensor nodes for covering robot parts, which need a precise tactile sense such as hands. However, some robot parts such as its back do not need an accurate tactile sense. Thus, the parallel usage of high-performance integrated capacitive sensors for small sensitive areas, commercially available capacitive tactile sensors for large sensitive areas and low cost resistive tactile sensors [17] for insensitive areas is a reasonable solution in terms of system balance.

For implementing this solution, we designed a multiple sensor platform with a dedicated CMOS-LSI (referred to as “sensor platform LSI”), in which an on-chip clock generator (typical frequency is 64 MHz), an on-chip diode-based temperature sensor, sensor interfaces, signal processing and communication components such as capacitance-to-frequency converter, frequency counter, resistance-to-voltage converter, voltage-to-digital converter (in general, analog-to-digital converter: ADC), memory, data transmitting and commands receiving circuits are embedded. We have also used unique data and command packet formats for the developed asynchronous bus-based serial communication method [18,19]. Considering system requirements, parameters of the sensor platform LSI such as sensing mode, sensitivity and sampling rate can be configured flexibly. Our previous paper [20] has already shown some basic function test results of the sensor platform LSI.

This paper first proposes the concept of our serial bus-based tactile sensor network system with multiple kinds of sensors. Then, the fundamental functions of the sensor platform LSI such as capacitance-to-digital and resistance-to-digital converters were evaluated. Finally, the multi-sensor system was demonstrated as a framework of our proposed serial bus-based tactile sensor network system with the sensor platform LSIs by simultaneous utilization of discrete capacitive and resistive type force sensors and embedded temperature sensors.

This paper is organized as follows: Section 2 shows our system overview and function details. Section 3 explains function evaluations. Section 4 gives system implementation considering practical use. Section 5 concludes this paper.

## 2. Tactile Sensor Network System

### 2.1. System Overview

**Figure 2:**
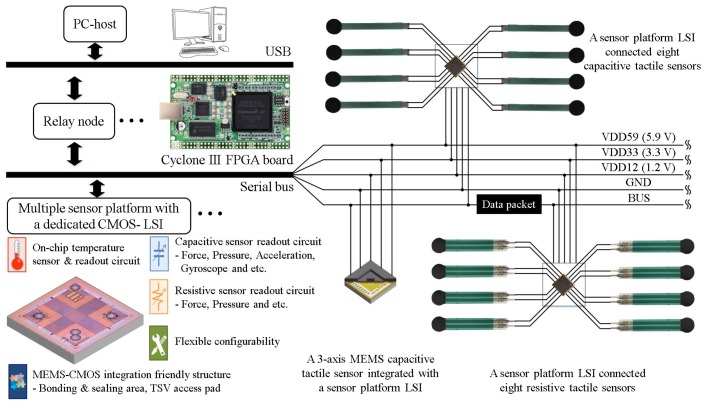
Proposed serial bus-based tactile sensor network system with multiple sensors using the sensor platform LSIs (Large-Scale Integration) on a shared bus line.

Figure 2 shows the overview of the sensor system we propose. Each sensor platform LSI can be configured for operating at either capacitive or resistive sensing mode. In the capacitive sensing mode, the sensor platform LSI processes signals from eight capacitance channels. Each capacitance channel can connect a capacitive type sensor. These eight capacitance channels can also be used for a three-axis MEMS capacitive sensor [21]. In the resistive sensing mode, the sensor platform LSI processes signals from eight resistance channels. Each resistance channel can connect a resistive type sensor. Data packets of both sensing modes also include temperature sensing data from the on-chip temperature sensor.

Sensor platform LSIs are mounted on a flexible cable that includes five lines, three power lines (VDD12, VDD33, VDD59), one signal line (BUS) and one ground line (GND). Stretchable and bendable cables can be used for covering curved and movable robot surfaces [14]. The cable is connected to an FPGA-based relay node. The relay node receives data packets from the sensor platform LSIs for some primary data processing such as data decoding and error check with Cyclic Redundancy Check (CRC) codes, and then transmits CRC passed data packets to the PC-based host. The relay node also receives configuration command packets from the host, and after encoding, transmits these command packets to a specific or all sensor platform LSIs. Because each sensor platform LSI has an 8-bit physical address for specific configuration, a relay node can handle 255 sensor platform LSIs at the maximum, and the address with all bits being 1 is used for broadcast. A USB interface (FT2232HL chip from Future Technology Devices International Ltd., Glasgow, UK) is used for communication between the relay node and the host. We assume up to 127 USB connections of the relay node for one host. As a result, more than 30,000 sensor platform LSIs in this system can be installed.

The host programmed by C# language analyzes data packets received from relay nodes and sends configuration command packets for sensor platform LSIs through relay nodes. Between the host C# software and the FT2232HL chip on the relay node, a Dynamic Link Library (DLL) file provided by Future Technology Devices International Ltd. is used for USB communication. For effective data communication, we proposed well customized variable length packet formats.

### 2.2. Multiple Sensor Platform with a Dedicated CMOS-LSI (Sensor Platform LSI)

**Figure 3:**
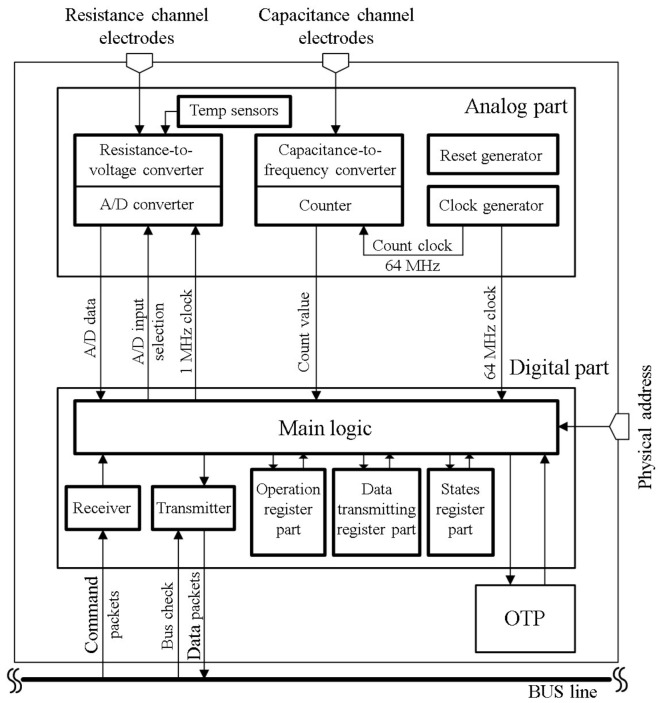
Block diagram of sensor platform LSI.

The sensor platform LSI was fabricated by a TSMC (Taiwan Semiconductor Manufacturing Co., Ltd., Hsinchu, Taiwan) 0.13 μm standard CMOS process technology. As shown in Figure 3, the analog part includes a clock generator, a reset generator, capacitance-to-frequency converter and resistance-to-voltage converter circuits and a temperature sensor. The on-chip clock is generated by a ring oscillator circuit, and its typical frequency is 64 MHz at design stage (about 60 MHz at measurement stage).

The digital part includes main logic, sensor readout, data transmitting and command receiving circuits, and three function register blocks. A nonvolatile One-Time Programmable (OTP) memory can store configuration information of the sensor platform LSI such as its own ID number for each chip separation, threshold values and sampling time. Voltage levels of physical address pads can be adjusted to set the ID information of a sensor platform LSI. We can choose to use ID information either from the OTP or the LSI pads. In the main logic, to reduce data packets for overcoming data congestion problem, we carried out the threshold and adaptation functions inspired by human tactile receptors [22].

#### 2.2.1. Capacitance-to-Frequency Converter

**Figure 4:**
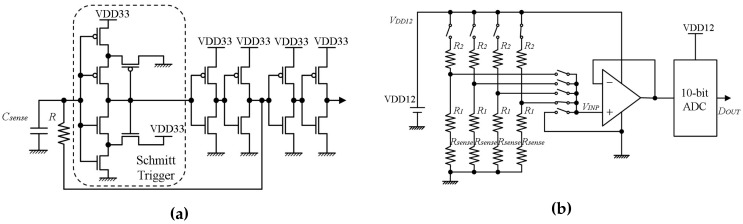
Diagrams of analog-to-digital converter circuits: (**a**) capacitance-to-frequency converter circuit; (**b**) resistance-to-voltage converter circuit.

As shown in Figure 4a, the capacitance-to-frequency converter circuit is based on a resistor-capacitor oscillator with a Schmitt-trigger circuit [23]. The converter circuit frequency *f* [Hz] is determined by its RC time constant, and its two designed threshold values ($V_{TH}$ [V], $V_{TL}$ [V]). The frequency value equals

(1) $$f=\frac{1}{R\;\cdot \;C_{sense}\;\cdot \;\left(2\left(\ln V_{TH}-\ln V_{TL}\right)\right)}=\frac{1}{R\;\cdot \;k_{c}\;\cdot \;C_{sense}},$$

where constant $k_{c}$ is determined by Schmitt-trigger circuit threshold values. $C_{sense}$ [F] is the capacitance value from a capacitance channel. Considering the parasitic capacitance $C_{c}$ [F], the frequency value equals

(2) $$f=\frac{1}{R\;\cdot \;k_{c}\;\cdot \;\left(C_{sense}+C_{c}\right)}.$$

The sampling time $T_{s}$ [s], which is based on the number of on-chip clock cycles of the sensor platform LSI, can be adjusted by configuration. In a certain sampling time, the count value $N_{c}$ equals

(3) $$N_{c}=T_{s}\;\cdot \;f.$$

Combining Equations (2) and (3), the relationship between the capacitance value from one capacitance channel and the count value is

(4) $$N_{c}=T_{s}\;\cdot \;\frac{1}{R\;\cdot \;k_{c}\;\cdot \;\left(C_{sense}+C_{c}\right)}.$$

#### 2.2.2. Resistance-to-Voltage Converter

Figure 4b illustrates the resistance-to-voltage converter circuit. Please note that, in Figure 4b, only four channels are drawn for explanation. According to the analog-to-digital input selection signal, one resistance channel is connected to the circuit at a time. The voltage $V_{INP}$ [V] equals

(5) $$V_{INP}=V_{DD12}\;\cdot \;\left(1-\frac{R_{2}}{R_{1}+R_{2}+R_{sense}}\right).$$

Through a 10-bit Successive Approximation (SAR) Analog-to-Digital Converter (ADC), the digital output $D_{OUT}$ is

(6) $$D_{OUT}=k_{r}\;\cdot \;V_{INP},$$

where constant $k_{r}$ is determined by the ADC. Combining Equations (5) and (6), the relationship between the resistance of the resistive sensor and its digital value is

(7) $$D_{OUT}=k_{r}\;\cdot \;V_{DD12}\;\cdot \;\left(1-\frac{R_{2}}{R_{1}+R_{2}+R_{sense}}\right).$$

## 3. Sensor Platform LSI Evaluation Results

**Figure 5:**
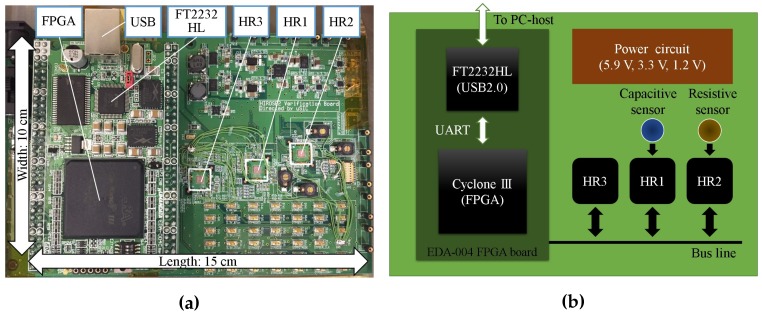
Evaluation board for sensor platform LSI test: (**a**) photograph; (**b**) schematic diagram.

As shown in Figure 5, we fabricated a Printed Circuit Board (PCB) to evaluate the sensor platform LSI. On this evaluation board, three sensor platform LSIs (HR1, HR2 and HR3) have been implemented and connected to a relay node through the same bus line. The relay node is based on a USB-FPGA board (EDA-004, HuMANDATA Ltd., Osaka, Japan). For the evaluation, we first used several capacitors and resistors with different nominal values to evaluate the performance of the capacitance-to-frequency converter and resistance-to-voltage converter in comparison with our design. Then, we used this system to evaluate the performance of commercially available capacitive sensors from Oga Inc. (Toyama, Japan) and resistive sensors from Interlink Electronics Inc. (Westlake Village, CA, USA).

### 3.1. Capacitance-to-Frequency Converter Evaluation with Fixed Capacitors

**Figure 6:**
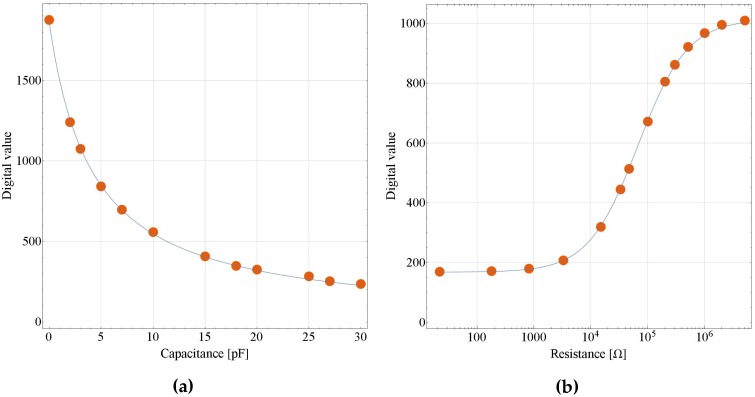
Curve fitting diagrams of measurement values: (**a**) relationship between capacitors’ nominal capacitance values and corresponding digital count values; (**b**) relationship between resistors’ nominal resistance values and corresponding digital values.

In Equation (4), after configuration, sampling time $T_{s}$ [s] can be regarded as a constant. We can obtain the value of $T_{s}/\left(R\;\cdot \;k_{c}\right)$ by curve fitting to points of nominal capacitance values of capacitors and their corresponding count value $N_{c}$. Wolfram Mathematica (Wolfram Research, Inc., Champaign, IL, USA) and the Levenberg–Marquardt algorithm were used for curve fitting, and Figure 6a shows the fitting result. The digital value of each point represents the average of more than 1000 measurement values. R-squared is 0.99987, which means the fitting explains 99.987% of the total variation in the data, which is about the average. The difference between the fitting and the design values of $T_{s}/\left(R\;\cdot \;k_{c}\right)$ is 4.89% of the design value.

### 3.2. Resistance-to-Voltage Converter Evaluation with Fixed Resistors

In Equation (7), $k_{r}\;\cdot \;V_{DD12}$ equals the maximum digital value $D_{max}$, which can be obtained when there is no resistor connected to the circuit. By curve fitting to points of nominal resistance values of resistors and their corresponding $D_{OUT}$ values, $R_{1}$ and $R_{2}$ can be achieved. As shown in Figure 6b, R-squared is 0.99995, which means the fitting explains 99.995% of the total variation in the data, which is about the average. The difference between the fitting and the design values of $R_{1}$ and $R_{2}$ are 11.03% and 11.62% of the design values, respectively.

### 3.3. Sensor Converter Evaluation with Sensors

**Figure 7:**
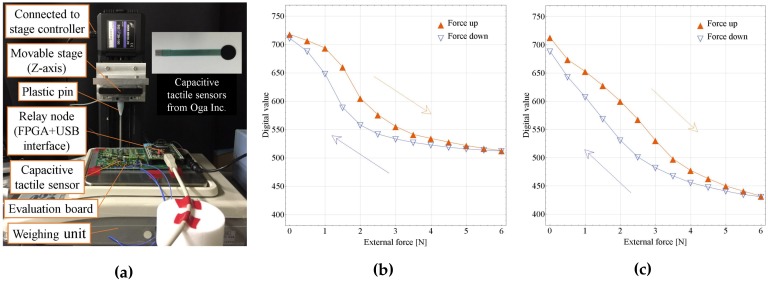
Capacitive tactile sensor evaluation: (**a**) evaluation setup; (**b**) relationship between external forces and decoded sensor outputs, when the pin directly contacts the sensor; (**c**) relationship between external forces and decoded sensor outputs, when the pin contacts the Polydimethylsiloxane (PDMS) cover on sensor.

**Figure 8:**
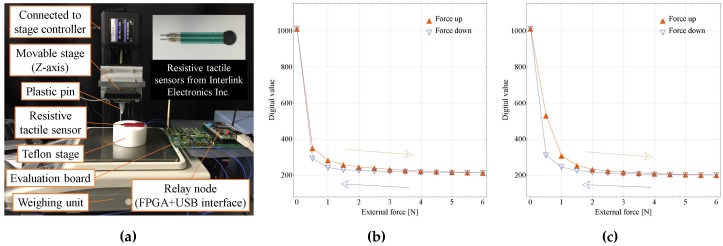
Resistive tactile sensor evaluation: (**a**) evaluation setup; (**b**) relationship between external forces and decoded sensor outputs, when the pin directly contacts the sensor; (**c**) relationship between external forces and decoded sensor outputs, when the pin contacts the PDMS cover on sensor.

As shown in Figure 7a and Figure 8a, to evaluate capacitive and resistive tactile sensors, a capacitive tactile sensor was connected to a sensor platform LSI (HR1), and a resistive tactile sensor was connected to another sensor platform LSI (HR2). By operating the movable stage in the *z*-axis (Mark-204-MS, SIGMA KOKI Co., Ltd., Tokyo, Japan), the external force can be applied on the sensor through a plastic pin. This force can be measured by a weighing unit (GF-6100, A&D Co., Ltd., Tokyo, Japan). After configuring the sensor platform LSI to the capacitive or resistive sensing mode and applying the external force, a large amount of sensing data can be analyzed by the C# software on the PC-host side. In the case of the capacitive sensor test, to reduce parasitic capacitance, the capacitive tactile sensor was mounted on the evaluation board near the sensor platform LSI.

Figure 7b,c shows external forces and decoded sensor output relationships of the capacitive sensor. The digital value of each point was the average of more than 1000 measurement values. Hysteresis loops of external forces from 0 N to 6 N were confirmed [17]. In the case of Figure 7c, a 2 mm thick, around 6 mm diameter Polydimethylsiloxane (PDMS) cover was put on the sensor for confirming one kind of usage situation. As shown in Figure 7b, an inflection point appears. We think the reason is that the relationship between the external forces and the average displacement between two plates of the capacitive sensor becomes nonlinear, with the relatively bigger sensor (Diameter: 8 mm) being pushed by a small pin (Diameter: 3 mm). This can be verified by Figure 7c. The PDMS cover distributes the external force and results in a more linear force and average displacement relationship, which leads to a more linear relationship between external forces and decoded sensor outputs. However, using the PDMS cover, the hysteresis becomes larger.

Figure 8b,c shows relationships between external forces and decoded sensor outputs of the resistive sensor. The digital value of each point was the average of more than 1000 measurement values. Hysteresis loops of external forces from 0 N to 6 N were confirmed [24]. In the case of Figure 8c, a 2 mm thick, around 6 mm diameter PDMS cover was put on the sensor. The adding of the PDMS cover resulted in a more moderate resistance change along with force change. However, the same as the capacitive sensor case, using the PDMS cover leads to a larger hysteresis.

## 4. Experiments on a Real-Time Data Acquisition System

### 4.1. Evaluation of a System with Multiple Sensors and Rigid Evaluation Board

**Figure 9:**
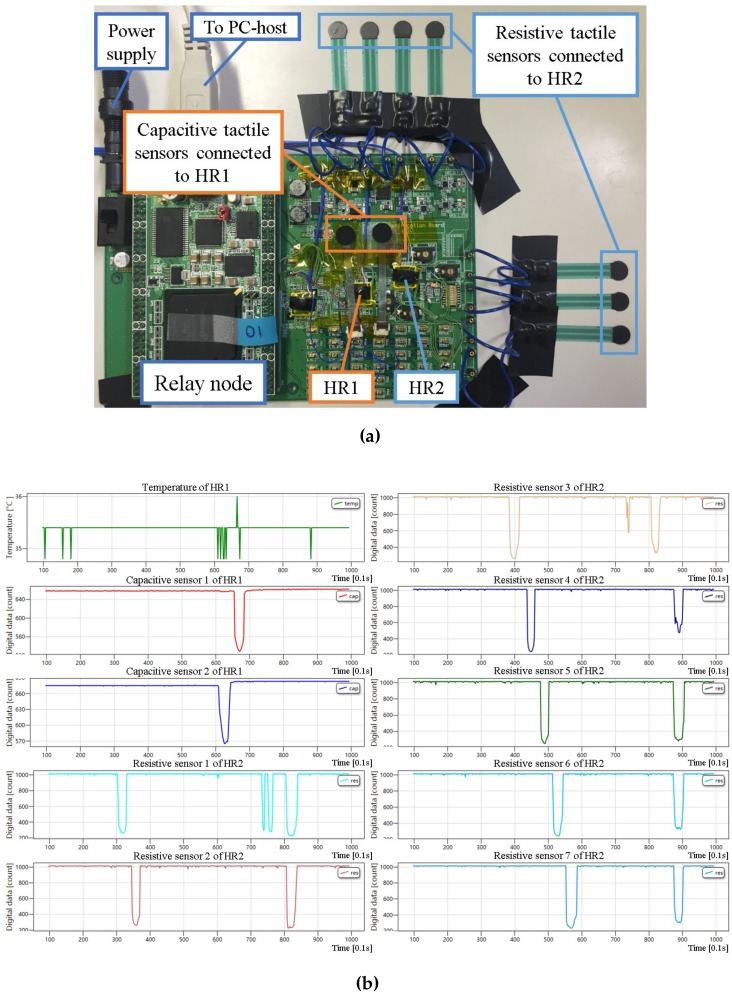
Simultaneous operation demonstration of two sensor platform LSIs with a temperature sensor, capacitive and resistive tactile sensors: (**a**) demonstration setup; (**b**) display of real-time multiple sensing data acquisition in C# software.

After evaluating the sensor platform LSI, we demonstrated our multi-sensor system. Figure 9 shows the demonstration setup and the display of the C# software on the PC-host with the previously used evaluation board. In this demonstration, two sensor platform LSIs, one relay node and one host were used. One of two sensor platform LSIs (HR1) was configured for capacitive sensing mode, and connected two capacitive sensors. The other sensor platform LSI (HR2) was configured for resistive sensing mode, and connected seven resistive sensors. These two sensor platform LSIs were connected to the relay node through the same bus. The C# software was used to display real-time sensing data of these nine tactile sensors as well as one temperature sensor from HR1. Digital values of each sensor output were updated every 0.1 s. As shown in Figure 9b, temperature sensing data by using the on-chip temperature sensor, tactile sensing data by using off-chip capacitive and resistive sensors were acquired simultaneously.

### 4.2. Evaluation of Multi-Sensor System with Flexible Cable Connection

After demonstrating our multi-sensor system on the evaluation board, we implemented more sensor platform LSIs on one bus line by using Flexible Printed Circuits (FPC) cables for practical robot applications.

**Figure 10:**
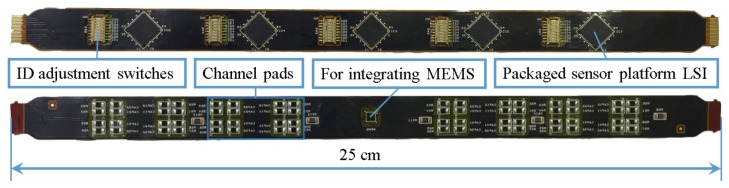
Photograph of Flexible Printed Circuits (FPC) cable with five sensor platform LSIs.

As shown in Figure 10, five sensor platform LSIs are implemented in one cable. Sensor platform LSIs and ID switches are mounted on one side of the cable. On the other side of the cable, resistive and capacitive channel pads are mounted. These pads are used to connect *z*-axis resistive or capacitive tactile sensors by soldering. The middle sensor platform LSI is prepared for integrating with a MEMS capacitive sensor, so there is no soldering pad connected to it. A connector was used to connect the ends of cables.

**Figure 11:**
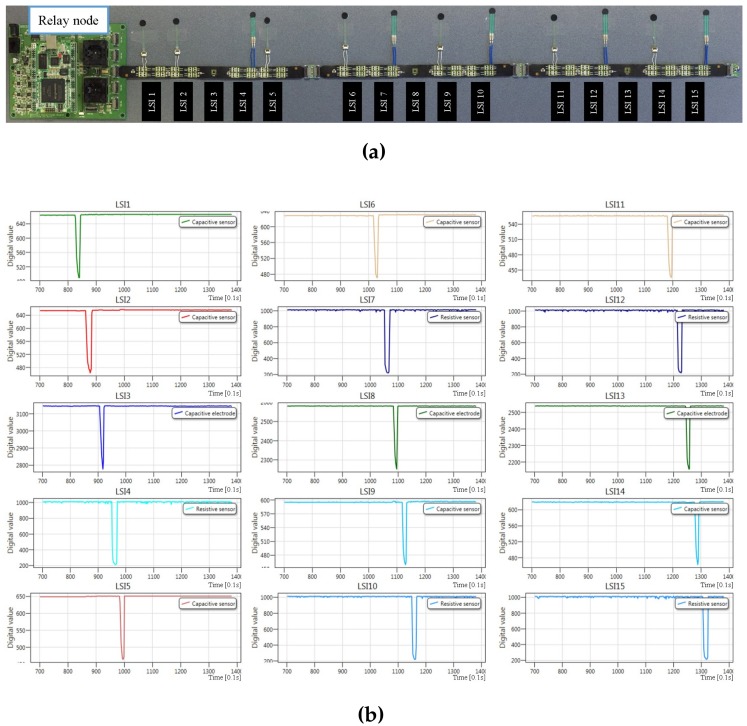
Simultaneous operation demonstration of 15 sensor platform LSIs configured for capacitive or resistive sensing mode: (**a**) demonstration setup, Nos. 1, 2, 5, 6, 9, 11, 14 sensor platform LSIs connect one capacitive tactile sensor each and Nos. 4, 7, 10, 12, 15 sensor platform LSIs connect one resistive tactile sensor each; (**b**) display of real-time multiple sensing data acquisition in C# software.

Figure 11a shows a multi-sensor system demonstration setup with 15 sensor platform LSIs. In this demonstration, three FPC cables were connected in series to a relay node. Among these sensor platform LSIs, seven were configured for capacitive sensing mode, and each of them connected one *z*-axis capacitive tactile sensor. Another five sensor platform LSIs were configured for resistive sensing mode, and each of them connected one *z*-axis resistive tactile sensor. Although each sensor platform LSI can connect eight capacitive or resistive *z*-axis tactile sensors, we only connected one tactile sensor to show the LSI’s operation on one bus line. For the sensor platform LSI at the middle position of each cable, since there are only small sized capacitive MEMS sensor connection pads, we touched these exposed pads by fingers to check whether they were operating. Fingers have several hundreds of picofarads capacitances, so the three middle sensor platform LSIs were configured for capacitive sensing mode. Figure 11b depicts the C# software display on the PC-host, which shows the touch on the sensor or exposed pads of each sensor platform LSI. This demonstration shows the simultaneous operation of 15 sensor platform LSIs with two kinds of tactile sensors, capacitive and resistive tactile sensors.

**Figure 12:**
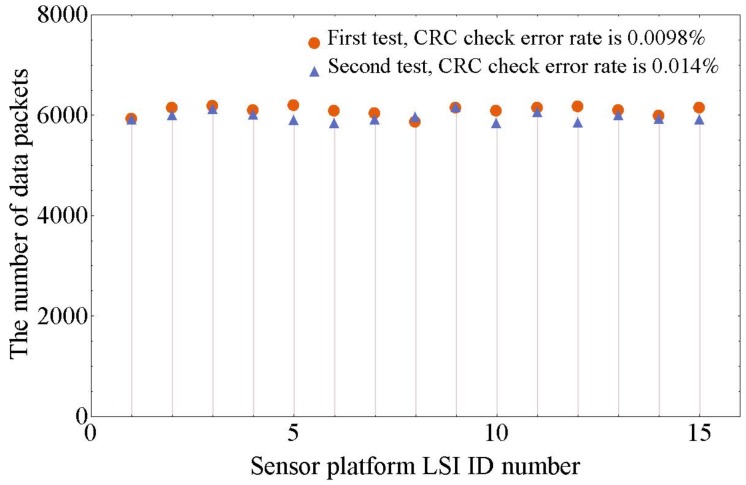
Data receiving results when 15 sensor platform LSIs were operating simultaneously.

Figure 12 shows the data receiving results at the PC-host side of two tests. In each test, three FPC cables were connected in series to a relay node. All of the 15 sensor platform LSIs were configured for capacitive sensing mode and operating simultaneously. The data sending rate of each sensor platform LSI was set to 1 Mbps, and the data receiving time at PC-host was 40 s in each test. To relieve data congestion, the adaption function [22] was turned on by configuration. As shown in Figure 12, the CRC check error rates are 0.0098% and 0.014%. These CRC check error data packets come from the data collision on the bus. The maximum difference of the number of data packets received from different sensor platform LSIs in these two tests are 5.6% and 5.4%, respectively. All of the 120 capacitive channels are sampled at a frequency of about 146 Hz. These results have proved that the multi-sensor system can acquire numerous sensing data with low data loss and similar data transmission frequency from every sensor platform LSI on the shared bus.

For the demonstrated multi-sensor system with 15 sensor platform LSIs, from Figure 12, the calculated sampling frequency is 146 Hz (the minimum of the number of received data packets 5859 divided by 40 s), which is much higher than the systems shown in the Table 1. There are eight capacitance-to-digital converters in each sensor platform LSI. In all, there are 120 capacitance-to-digital converters in this system. The length of the bus line is 75 cm, which shows an ability to cover larger areas. Since each sensor platform LSI can connect eight *z*-axis tactile sensors, the demonstrated multi-sensor system can include 120 *z*-axis tactile sensors.

Note that the demonstrated multi-sensor system is a framework of our proposed serial bus-based tactile sensor network system. This paper confirmed the structure of the system and functions of the sensor platform LSI, which is an important milestone of our project.

## 5. Conclusions

In this paper, we present the concept of our serial bus-based tactile sensor network system with the sensor platform LSI. Three types of sensors—temperature sensors, capacitive and resistive tactile sensors—can be included in this sensor system. An evaluation board was fabricated to test basic functions of the sensor platform LSI such as the capacitance-to-digital and resistance-to-digital converters, which function well as designed. Then, based on the evaluation board, we demonstrated the multi-sensor system by using one PC-host, one relay node, two sensor platform LSIs and nine tactile sensors, which showed the implementation of the multi-sensor system with three kinds of sensors—on-chip temperature sensor, off-chip capacitive and resistive tactile sensors. Finally, we used the FPC cables to demonstrate our multi-sensor system with one PC-host, one relay node, 15 sensor platform LSIs and 12 tactile sensors for real robot use. In conclusion, the multi-sensor system with up to 15 sensor platform LSIs on a bus line supporting temperature, capacitive and resistive sensing was successfully demonstrated. As a next step, we will install more sensor platform LSIs in the multi-sensor system and measure its features related to the data congestion and collision on the shared bus.

## Figures and Tables

**Table 1:** Summary of some tactile sensor systems with their performances.

System Structure	Sensor Output Digitization Place	Force Sensing Principle	The Number of Capacitors or Resistors	Covering Area	The Number of Analog-to-Digital Converters (ADC)	Sampling Frequency (Hz)	Ref.
Tactile sensor array with scanning readout circuitry	Off the sensor array	Capacitive	8×8	1.6×1.6 ${\mathrm{cm}}^{2}$	1 (AD7153)	3	[1]
			8×8	3.6×3.6 ${\mathrm{cm}}^{2}$	1 (ADuC841)	Not mentioned	[2]
			16×16	2.2×2.2 ${\mathrm{cm}}^{2}$	1	20	[3]
		Resistive	8×8	4×4 ${\mathrm{cm}}^{2}$	1 (PIC18F2523)	16	[4]
			8×12	9×13 ${\mathrm{cm}}^{2}$	1 (dsPIC30F5015)	Not mentioned	[5]
Serial bus-based tactile sensor network	At the place of sensation	Capacitive	12×16	3.9×16 ${\mathrm{cm}}^{2}$	16 (AD7147)	25	[10]
			114	Not mentioned	114 (MPL115A2)	50	[11]
		Capacitive or resistive plus temperature sensing	8×15 (Potentially, more than 15 LSIs can be connected.)	75 cm (bus line length)	8×15	146	**This work**

## Supplementary Materials

Supplementary File 1
